# *Akkermansia muciniphila* MucT attenuates sodium valproate‐induced hepatotoxicity and upregulation of *Akkermansia muciniphila* in rats

**DOI:** 10.1111/jcmm.18026

**Published:** 2023-11-14

**Authors:** Zhi Cui, Liang Xu, Ming Zhao, Luping Zhou

**Affiliations:** ^1^ Zhejiang Cancer Hospital, Hangzhou Institute of Medicine (HIM) Chinese Academy of Sciences Hangzhou China; ^2^ Department of Orthopaedics of the 3rd Xiangya Hospital Central South University Changsha China

**Keywords:** *Akkermansia muciniphila*, endoplasmic reticulum stress, gut barrier function, hepatotoxicity, sodium valproate

## Abstract

In the previous study, we found that the oral sodium valproate (SVP) increased the relative abundance of *Akkermansia muciniphila* (*A. muciniphila*) in rats, and plasma aspartate transaminase (AST) and alanine aminotransferase (ALT) activities were positively correlated with *A. muciniphila* levels. This study aimed to further investigate the role of *A. muciniphila* in SVP‐induced hepatotoxicity by orally supplementing rats with the representative strain of *A. muciniphila*, *A. muciniphila* MucT. Additionally, the fresh faeces were incubated anaerobically with SVP to investigate the effect of SVP on faecal *A. muciniphila* in the absence of host influence. Results showed that *A. muciniphila MucT* ameliorated the hepatotoxicity and upregulation of *A. muciniphila* induced by SVP. SVP also induced a noteworthy elevation of *A. muciniphila* level in vitro, supporting the observation in vivo. Therefore, we speculate that *A. muciniphila* MucT may be a potential therapeutic strategy for SVP‐induced hepatotoxicity. In addition, the increased *A. muciniphila* induced by SVP may differ from *A. muciniphila* MucT, but further evidence is needed. These findings provide new insights into the relationships between *A. muciniphila* and SVP‐induced hepatotoxicity, highlighting the potential for different *A. muciniphila* strains to have distinct or even opposing effects on SVP‐induced hepatotoxicity.

## 1 INTRODUCTION

Sodium valproate (SVP) is a widely prescribed antiepileptic drug and is also used to treat convulsions, migraines and bipolar disorders in adults.^1^ However, adverse reactions are commonly associated with SVP use, with hepatotoxicity being one of the severe adverse reactions. Hepatotoxicity has been plaguing the clinical applications of SVP, which can progress to acute liver failure and even death in severe cases.^2^ In March 2014, the United States Food and Drug Administration issued a black box warning for the hepatotoxicity associated with STAVZOR (valproic acid delayed‐release capsules). Despite its associated risks, SVP continues to be widely used due to its low cost and good efficacy. Some measures, such as strictly grasping the indications and dosage, and regularly checking liver function, help reduce the risk of hepatotoxicity induced by SVP, but it is not enough. These underscore the need for a comprehensive investigation into the pathogenesis of SVP‐induced hepatotoxicity and the identification of novel therapeutic targets and early warning indicators.

As a newly recognized functional organ, gut microbiota plays an important role in physiological regulation and disease development, and is regarded as the ‘second genome’ of human beings.^3^ The gut microbiota is a dense and diverse group of microorganisms containing bacteria, fungi, viruses, archaea, protozoa and phages.^4^, ^5^ Recent studies have reported that gut microbiota is involved in the hepatotoxicity of various substances, such as atrazine,^6^ 1,4‐dioxane,^7^ difenoconazole,^8^ geniposide,^9^ palmitic acid,^10^ acetaminophen,^11^ tacrine,^12^ polychlorinated biphenyl 126^13^ and N,N‐dimethylformamide.^14^ The alteration of the gut microbiota, eventuated from exogenous substances, causes dysfunction of the gut microbiota and intestinal tract, and then partially mediates the hepatotoxicity of exogenous substances. The study of the ‘drug‐microbe‐gut‐liver’ model and the interactions of its components is of great significance, and it is becoming a new direction for the exploration of the mechanism of drug‐induced liver injury. Our previous study demonstrated that SVP resulted in a disruption of the gut microbiota's structure with a 140‐fold increase in the relative abundance of faecal *Akkermansia muciniphila* (*A. muciniphila*) in rats, which was partially ameliorated by ginsenoside compound K, a liver‐protective compound.^15^

*A. muciniphila* is a gut symbiotic bacterium that colonizes the mucosal layer of the intestines and utilizes mucin as its sole source of carbon, nitrogen, and energy. This bacterium is highly valued for its ability to modulate metabolism, regulate immune responses and maintain gut health.^16^, ^17^ It is considered as an important candidate for the development of next‐generation probiotics and has a protective effect against several liver diseases, such as metabolic dysfunction‐associated fatty liver disease (Formerly known as nonalcoholic fatty liver disease),^18^ immune‐mediated liver injury,^19^ acetaminophen‐mediated hepatotoxicity,^20^ high‐fat diet/carbon tetrachloride‐induced liver injury^21^ and alcoholic liver disease.^22^ However, some studies have suggested that the increase in its relative abundance may not always be beneficial. For example, in male mice, high L‐carnitine ingestion has been shown to lead to an increase in the relative abundance of *A. muciniphila*, which was strongly and positively correlated with aspartate transaminase (AST), interleukin 1, tumour necrosis factor α, tumour necrosis factor β and malondialdehyde (MDA) levels.^23^ In both specific pathogen‐free and germ‐free *IL10* ^−/−^ mice, *A. muciniphila* was found to promote intestinal inflammation and act as a pathobiont to exacerbate colitis in a genetically susceptible host.^24^ Additionally, *A. muciniphila* contributes to enhancing the pathogen susceptibility of *Citrobacter rodentium* and *Clostridium difficile*,^25^, ^26^ and dietary haem‐induced epithelial hyperproliferation.^27^ These findings suggest that *A. muciniphila* may exhibit a diverse effect under different conditions, thus necessitating an in‐depth investigation of the role of *A. muciniphila* in specific conditions.

Therefore, further investigation is necessary to determine whether *A. muciniphila* exacerbates or ameliorates SVP‐induced hepatotoxicity. In this study, we focused on the effect and mechanism of *A. muciniphila* MucT, a widely studied representative strain of *A. muciniphila*, on SVP‐induced hepatotoxicity and verified the impact of SVP on the relative abundance of *A. muciniphila* in vitro and in vivo.

## 2 MATERIALS AND METHODS

### 2.1 Animal experiment

Male Sprague–Dawley rats (Weighing 150–200 g) were obtained from the Beijing Vital River Laboratory Animal Technology Co., Ltd, China (NO. 110011221106571875) and maintained under specific pathogen‐free conditions. The rats were kept in an environment with a temperature range of 20–22°C and a humidity level of 70%–75%. Additionally, they were subjected to a 12‐hour light–dark cycle and provided ad libitum access to water and rat maintenance feed. The standard commercial rat maintenance feed (21.5% protein, 67.4% carbohydrates and 11.1% fat) was purchased from the Xietong Pharmaceutical Bio‐engineering Co., Ltd. (Jiangsu, China). After 1 week of adaptive feeding, the rats weighed between 200 and 254 g. All rats were randomly assigned into one of four groups (*n* = 5 per group): sterile anaerobic phosphate buffer solution (PBS) control group (Con), *A. muciniphila* MucT control group (MucT), SVP group, and combined *A. muciniphila* MucT and SVP group (MucT + SVP). *A. muciniphila* MucT was orally gavaged with 10^8^ colony‐forming units (CFU) in 1 mL sterile anaerobic PBS once every other day for 15 days, which was set by comprehensively considering the actual situation of this study and publications.^18^, ^20^ SVP was orally administered at 600 mg/kg for 15 days,^28^, ^29^ one dose instead of two doses to avoid SVP meeting oral *A. muciniphila* MucT too soon. The time and dosage of administration were as follows:

**Table:** 

Group	Administration (intragastric)	
	9:00 AM. (1 mL)	5:00 PM. (5 mL/kg)
Con	Sterile anaerobic PBS	Sterile anaerobic PBS
MucT	10^8^ CFU/1 mL MucT	Sterile anaerobic PBS
SVP	Sterile anaerobic PBS	600 mg/kg SVP
MucT + SVP	10^8^ CFU/1 mL MucT	600 mg/kg SVP

Fresh faeces were collected from six specific pathogen‐free male Sprague–Dawley rats to investigate the impact of SVP on the level of *A. muciniphila* in vitro.

All animal care and use protocols were approved by the Laboratory Animal Ethics Committee of Servicebio (Servicebio animal (welfare) NO. 2022114) and conducted in strict accordance with the National Institute of Health Guide for the Care and Use of Laboratory Animals (NIH Publications NO. 80–23, revised 1996).

### 2.2 Sample Collection

All rats were weighed 24 hours after the final administration of SVP. At the time of sample collection, all rats had fasted for 12 hours. Blood and faecal samples were collected before euthanization, which was performed via inhalation of CO_2_ at a rate of 10%–30% of the chamber volume per minute. Plasma was separated from blood by refrigerated centrifugation (3000 rpm, 4°C, 10 min) and transferred to labelled storage tubes for biochemical assay. Following euthanization, the liver was immediately removed, rinsed in physiological saline, blotted dry with sterile gauze and weighed. Colon tissues were also harvested. A portion of the hepatic and colon tissues were immediately immersed in an appropriate fixative for follow‐up experiments, while another portion of the hepatic and colon tissues were quick‐frozen in liquid nitrogen and stored at −80°C for subsequent assays.

### 2.3 Assessment of hepatic index

The body weight and liver wet weight of each animal were recorded to calculate the hepatic index (Liver wet weight/body weight × 100%).

### 2.4 Plasma and liver biochemical parameter analysis

Plasma levels of alanine aminotransferase (ALT), AST and lactate dehydrogenase (LDH) were evaluated using a Hitachi 7180 automatic biochemistry analyzer (Hitachi, Japan). Liver tissues were rinsed with ice‐cold PBS to remove excess blood thoroughly and then minced into small pieces. The small tissue pieces were homogenized in ice‐cold PBS containing protease and phosphatase inhibitor cocktails (Weight of liver tissues: volume of PBS = 1:9) by a tissue grinder; the mixture was then frozen and thawed two times and finally centrifuged at 5000 g for 5 min to separate the supernatant. Hepatic MDA content, superoxide dismutase (SOD) activity, catalase (CAT) activity, glutathione peroxidase (GPx) activity and reduced glutathione (GSH) concentration in the supernatants were measured as described previously using commercially available kits (Nanjing Jiancheng Bioengineering Institute, China).^30^, ^31^, ^32^ The hepatic 8‐Hydroxy‐deoxyguanosine (8‐OHdG) content was assayed by the rat 8‐OHdG ELISA Kit (Wuhan Huamei Biotech Co., Ltd., China). Additionally, plasma level of lipopolysaccharide (LPS) was assessed using the rat LPS ELISA Kit (Dogesce, Beijing, China).

### 2.5 Detection of protein level

The supernatant of liver homogenate described in section ‘*2.4 Plasma and liver biochemical parameter analysis’* was used to detect cytochrome c (Cyt‐c) content in the cytoplasm by an ELISA Assay Kit (Dogesce, Beijing, China). Additionally, the liver tissues were crushed with a tissue grinder and lysed for 30 min with a RIPA lysis buffer containing protease and phosphatase inhibitor cocktails. After incubation, the samples were centrifuged at 12,000 rpm for 15 min at 4°C to separate the supernatant. The total protein concentration in the supernatant was analyzed using a bicinchoninic acid assay. As done previously, total protein was extracted and protein concentration determined.^33^ The supernatant containing total hepatic protein was used to detect the levels of hepatic mitochondrial respiratory chain complex 1 (Complex I), mitochondrial respiratory chain complex 4 (Complex IV), C/EBP‐homologous protein (Chop), 78 kDa glucose‐regulated protein (Grp‐78) and cysteinyl aspartate‐specific proteinase‐12 (Caspase‐12) through the ELISA Assay Kit (Dogesce, Beijing, China).

### 2.6 Haematoxylin–eosin and alcian blue staining

Liver and colon tissues were fixed in 4% paraformaldehyde for 24 h at room temperature. As the literature described, the specimens were dehydrated in ascending grades of alcohol solution (75% for 4 h, 85% for 2 h, 90% for 2 h, 95% for 1 h and 100% for 1 h), cleared in xylol and embedded in paraffin wax.^34^ Next, the paraffin‐embedded samples were cut into sections with a thickness of 3 μm. Subsequently, the paraffin‐embedded tissue sections were stained using the haematoxylin–eosin staining kit and alcian blue staining kit (Servicebio, Wuhan, China), and coverslipped in a fully automatic dyeing and coverslipping machine composed of a Tissue‐Tek Prisma® *Plus* stainer and Tissue‐Tek Film® Coverslipper (SAKURA, Japan). Images were acquired using a pathological section scanning system (Olympus, Japan) and analysed using the corresponding software to determine colonic mucosal thickness and crypt depth, 5 tests per sample. The histopathologist responsible for analysis was blinded to the experimental groups to minimize potential bias.

### 2.7 Anaerobic incubation of gut microbiota with sodium valproate

Fresh faecal samples were mixed with 10 mL anaerobic PBS and were centrifuged at 200 g for 5 min at 4°C, and this process was repeated twice. The resulting supernatants were combined and centrifuged at 300 *g* for 5 min at 4°C to remove the precipitate. The supernatants were then centrifuged at 8000 *g* for 10 min at 4°C to obtain the gut microbiota, which was resuspended in 1 mL anaerobic PBS. A total of 500 μL of this mixture was mixed with 20 mL of anaerobic microbial medium, with or without 10 mM SVP, and then incubated in an anaerobic incubator for 48 h. Following this, the bacteria were collected for bacterial DNA extraction.

### 2.8 Quantification of *Akkermansia muciniphila* abundance

Bacterial DNA was extracted using the Acmec Fecal Genomic DNA Extraction Kit (Acmec, Shanghai, China). The concentration and purity (A260/A280 and A260/A230 ratios) of the DNA samples were assessed using the NanoDrop One Microvolume UV–Vis Spectrophotometer (Thermo Fisher Scientific, USA). The qRT‐PCR amplification was performed using the ChamQ Universal SYBR qPCR Master Mix (Vazyme Biotech Co., Ltd., China) and CFX96 Real‐Time PCR System (Bio‐rad, USA). The PCR reaction was performed in a total volume of 10 μL, consisting of 5 μL 2 × ChamQ Universal SYBR qPCR Master Mix, 0.25 μL of forward primer (0.25 μM final), 0.25 μL of reverse primer (0.25 μM final) and 4.5 μL of diluted DNA template. The PCR program included a preliminary denaturation at 95°C for 3 min, followed by 40 amplification cycles of 95°C for 10 sec and 55°C for 30 sec, and finally a default dissolution curve program setting for the instrument. The relative abundance of *A. muciniphila* was calculated by using the following formula: 2^−ΔCt^ = 2^−(Ct of *A. muciniphila* − Ct of total bacteria)^.^35^ The sequences of the primers used for PCR are provided in the following table:

**Table:** 

Bacteria	Primer (5′‐3′)
Total bacteria (16S rDNA)	Forward, GTGYCAGCMGCCGCGGTAA
	Reverse, GGACTACNVGGGTWTCTAAT
*A. muciniphila*	Forward, CAGCACGTGAAGGTGGGGAC
	Reverse, CCTTGCGGTTGGCTTCAGAT

### 2.9 Statistical analysis

Statistical analysis was performed using GraphPad Prism 9.0.0 software. Data are presented as mean ± standard deviation. Differences in indices, except the relative abundance of *A. muciniphila* in the in vitro experiment, were assessed using a one‐way analysis of variance followed by the least significant difference test. Nonparametric tests were employed for data that did not conform to a normal distribution or did not have equal variances. The Wilcoxon matched‐pair signed‐rank test was used to compare the relative abundance of *A. muciniphila* between the two groups in the in vitro experiment. Statistical significance was defined as *p* < 0.05.

## 3 RESULTS

### 3.1 *Akkermansia muciniphila* MucT improved sodium valproate‐induced liver injury

**FIGURE 1:**
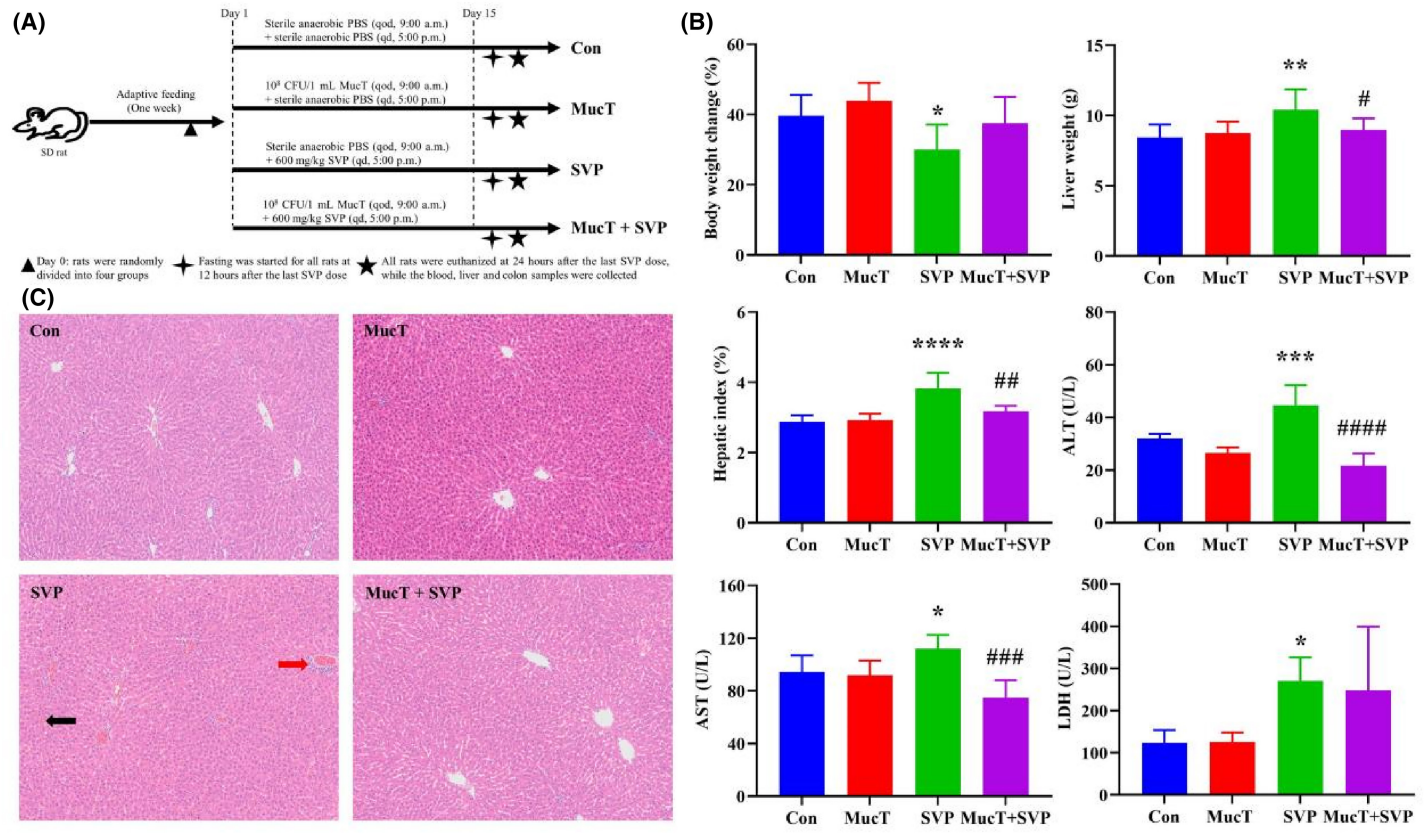
Effect of *Akkermansia muciniphila* MucT on sodium valproate‐induced liver injury. (A) Experimental design for the animal study. (B) Changes in body weight, liver weight, hepatic index and plasma levels of ALT, AST and LDH. Data are presented as mean ± standard deviation (*n* = 5). (C) Representative liver histology (HE staining of liver tissue, ×100). The SVP group displayed hepatic sinusoidal dilatation and congestion (Black arrow) as well as inflammatory cell infiltration (Red arrow). Con: sterile anaerobic phosphate buffer solution; MucT: *A. muciniphila* MucT control group (10^8^ CFU in 1 mL sterile anaerobic phosphate buffer solution, once every other day, 9:00 AM.); SVP: 600 mg/kg SVP, once daily, 5:00 PM.; MucT + SVP: combined *A. muciniphila* MucT and SVP group. Con, control; *A. muciniphila*, *Akkermansia muciniphila*; SVP, sodium valproate; ALT, alanine aminotransferase; AST, aspartate transaminase; LDH, lactate dehydrogenase. *p < 0.05; **p < 0.01; ***p < 0.001; ****p < 0.0001, compared with the control group; ^#^ *p* < 0.05; ^##^ *p* < 0.01; ^###^ *p* < 0.001; ^####^ *p* < 0.0001, compared with the SVP group (One‐way analysis of variance followed by the least significant difference test).

Liver injury was induced by administering SVP via gavage. The liver injury was characterized by elevated hepatic index (3.83 ± 0.44% vs 2.88 ± 0.18%, *p* < 0.0001), increased plasma ALT, AST and LDH levels (44.70 ± 7.55 U/L vs 32.02 ± 1.70 U/L, *p* < 0.001; 111.85 ± 10.52 U/L vs 94.22 ± 12.56 U/L, *p* < 0.05; 270.61 ± 55.06 U/L vs 123.26 ± 30.45 U/L, *p* < 0.05), hepatic sinusoidal dilatation, sinusoidal congestion and inflammatory cell infiltration. In contrast, treatment with *A. muciniphila* MucT significantly reduced the hepatic index and plasma ALT and AST levels by 16.97%, 51.45% and 33.22% (*p* < 0.01, < 0.0001, < 0.001), respectively. Moreover, there was only sporadic hepatic sinusoidal dilatation, sinusoidal congestion and inflammatory cell infiltration in the MucT + SVP group compared to that of the SVP group. These results are presented in Figure 1.

### 3.2 Effects on mitochondrial function, oxidative stress and endoplasmic reticulum stress biomarkers

Table 1 summarizes the detection results of mitochondrial function biomarkers in rat liver examined in this study, including the cytosolic Cyt‐c content, complex I and complex IV protein levels, and complex IV/complex I ratio. SVP administration led to a significant decrease in complex I protein levels (1.35 ± 0.38 vs 2.06 ± 0.69 ng/mgprot, *p* < 0.05) and a significant increase in complex IV/complex I ratio (0.30 ± 0.04 vs 0.24 ± 0.01, *p* < 0.05) and cytosolic Cyt‐c content (37.80 ± 6.48 vs 31.28 ± 3.99 nmol/gprot, *p* < 0.05). In contrast, the value of Cyt‐c content in the combined MucT and SVP group was significantly lower compared to the SVP group and was closer to that of the Con group. However, there were no significant differences in complex I protein level and complex IV/complex I ratio between the SVP and MucT + SVP groups.

**TABLE 1:** Effect of sodium valproate alone and combined with *Akkermansia muciniphila* MucT on mitochondrial function biomarkers in rat liver.

Group	Cyt‐c (nmol/gprot)	Complex I (ng/mgprot)	Complex IV (pg/mgprot)	Complex IV/Complex I
Con	31.28 ± 3.99	2.06 ± 0.69	498.96 ± 159.10	0.24 ± 0.01
MucT	30.57 ± 3.27	1.50 ± 0.54	367.03 ± 105.24	0.25 ± 0.05
SVP	37.80 ± 6.48*	1.35 ± 0.38*	401.22 ± 117.62	0.30 ± 0.04*
MucT + SVP	29.47 ± 2.73^##^	1.35 ± 0.26	391.49 ± 62.33	0.29 ± 0.02

*Note*: Con: sterile anaerobic phosphate buffer solution; MucT: *A. muciniphila* MucT control group (10^8^ CFU in 1 mL sterile anaerobic phosphate buffer solution, once every other day, 9:00 AM.); SVP: 600 mg/kg SVP, once daily, 5:00 PM.; MucT + SVP: combined *A. muciniphila* MucT and SVP group. Con, control; *A. muciniphila*, *Akkermansia muciniphila*; SVP, sodium valproate; Cyt‐c, cytochrome c; Complex I, mitochondrial respiratory chain complex 1; Complex IV, mitochondrial respiratory chain complex 4. *p < 0.05, compared with the control group; ^##^ *p* < 0.01, compared with the SVP group (One‐way analysis of variance followed by the least significant difference test).

**FIGURE 2:**
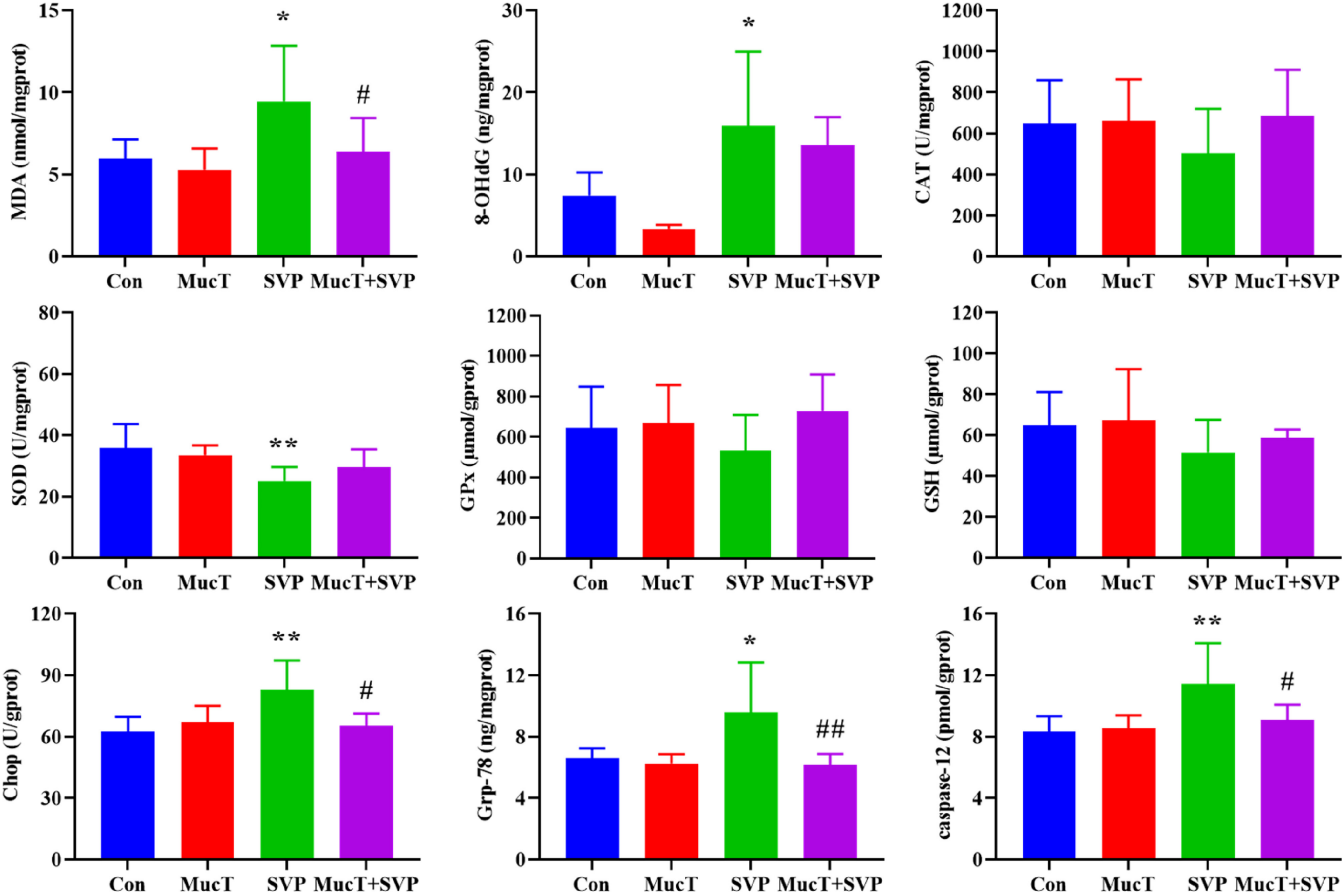
Effect of SVP alone and combined with *Akkermansia muciniphila* MucT on oxidative stress and endoplasmic reticulum stress biomarkers in rat liver. Oxidative stress biomarkers: MDA, 8‐OHdG, CAT, SOD, GPx and GSH. Endoplasmic reticulum stress biomarkers: Chop, Grp‐78 and caspase‐12. Con: sterile anaerobic phosphate buffer solution; MucT: *A. muciniphila* MucT control group (10^8^ CFU in 1 mL sterile anaerobic phosphate buffer solution, once every other day, 9:00 AM.); SVP: 600 mg/kg SVP, once daily, 5:00 PM.; MucT + SVP: combined *A. muciniphila* MucT and SVP group. Con, control; *A. muciniphila*, *Akkermansia muciniphila*; SVP, sodium valproate; MDA, malondialdehyde; 8‐OHdG, 8‐Hydroxy‐deoxyguanosine; CAT, catalase; SOD, superoxide dismutase; GPx, glutathione peroxidase; GSH, reduced glutathione; Chop, C/EBP‐homologous protein; Grp‐78, 78 kDa glucose‐regulated protein; caspase‐12, cysteinyl aspartate‐specific proteinase‐12. *p < 0.05; **p < 0.01, compared with the control group; ^#^ *p* < 0.05; ^##^ *p* < 0.01, compared with the SVP group (One‐way analysis of variance followed by the least significant difference test).

Figure 2 presents the levels of oxidative stress and endoplasmic reticulum stress biomarkers in liver tissue. MDA and 8‐OHdG are biomarkers for lipid peroxidation and oxidative DNA damage, respectively. The hepatic MDA and 8‐OHdG levels were significantly increased in the SVP group compared to the Con group (9.46 ± 3.39 vs 5.99 ± 1.14 nmol/mgprot, *p* < 0.05; 15.91 ± 9.08 vs 7.42 ± 2.82 ng/mgprot, *p* < 0.05). Notably, administration of *A. muciniphila* MucT effectively inhibited the increase in hepatic MDA content (6.37 ± 2.07 vs 9.46 ± 3.39 nmol/mgprot, *p* < 0.05), without obvious inhibition on the increase of hepatic 8‐OHdG level induced by SVP. In terms of hepatic antioxidant capacity, SVP administration significantly reduced SOD activity (25.06 ± 4.59 vs 35.75 ± 7.85 U/mgprot, *p* < 0.01) and led to a downward trend in CAT, GPx and GSH levels that was not significant. In contrast to the SVP group, the group treated with MucT + SVP also displayed only a trend of increased levels of CAT, GPx, SOD and GSH.

Compared to the control group, SVP administration resulted in a significant increase in the endoplasmic reticulum stress biomarkers including Chop, Grp‐78 and caspase‐12 protein levels (82.77 ± 14.46 vs 62.52 ± 7.11 U/gprot, *p* < 0.01; 9.55 ± 3.27 vs 6.57 ± 0.67 ng/mgprot, *p* < 0.05; 11.44 ± 2.65 vs 8.31 ± 1.02 pmol/gprot, *p* < 0.01). In the combined MucT and SVP group, there was a significant reduction in Chop, Grp‐78 and caspase‐12 levels by 21.08%, 35.60% and 20.77%, respectively (*p* < 0.05, < 0.01, < 0.05).

### 3.3 Effects on gut barrier function

**FIGURE 3:**
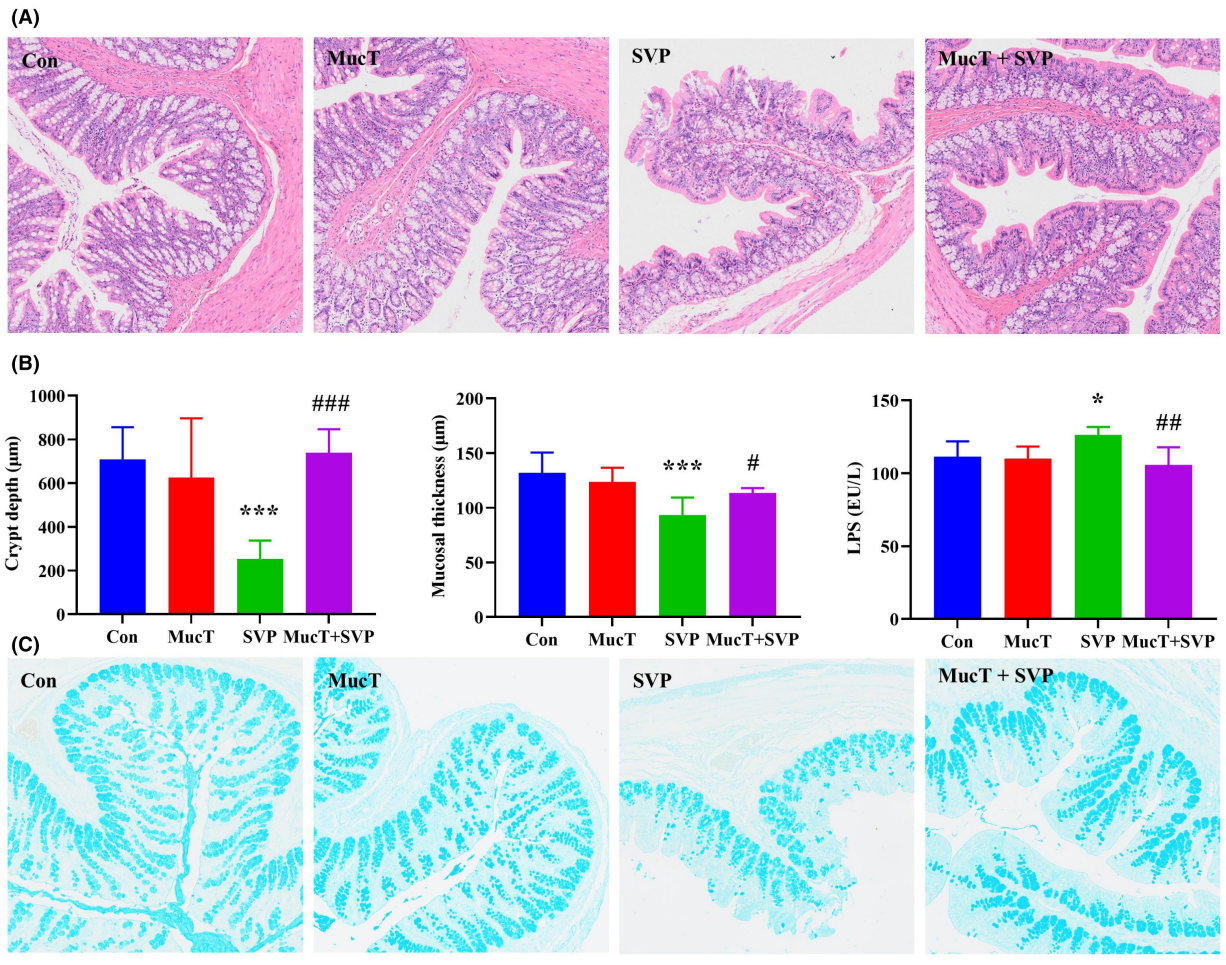
Effects of *Akkermansia muciniphila* MucT on the disruption of gut barrier integrity induced by sodium valproate. (A) Representative colon histology (HE staining of colon tissue, ×100). (B) Colonic crypt depth and mucosal thickness, plasma LPS level. Data are expressed as mean ± standard deviation (*n* = 5). (C) Alcian blue staining of colon tissue (×100). Con: sterile anaerobic phosphate buffer solution; MucT: *A. muciniphila* MucT control group (10^8^ CFU in 1 mL sterile anaerobic phosphate buffer solution, once every other day, 9:00 AM.); SVP: 600 mg/kg SVP, once daily, 5:00 PM.; MucT + SVP: combined *A. muciniphila* MucT and SVP group. Con, control; *A. muciniphila*, *Akkermansia muciniphila*; SVP, sodium valproate; LPS, lipopolysaccharide. *p < 0.05; ***p < 0.001, compared with the control group; ^#^ *p* < 0.05; ^##^ *p* < 0.01; ^###^ *p* < 0.001, compared with the SVP group (One‐way analysis of variance followed by the least significant difference test).

To assess intestinal barrier function, we analysed colonic histopathological changes, mucosal thickness, crypt depth, goblet cells and plasma LPS level (Figure 3). Compared to the control group, rats in the SVP group exhibited marked intestinal wall thinning and mucosal injury. Specifically, the colonic crypt depth and mucosal thickness in the SVP group decreased significantly by 64.23% and 29.25%, respectively (*p* < 0.001, < 0.001). These histopathological changes caused by SVP were significantly improved in the combined *A. muciniphila* MucT and SVP group. The results of alcian blue staining revealed a significant decrease in the number of goblet cells in response to SVP administration, but *A. muciniphila* MucT supplementation partially inhibited the loss of goblet cells. Furthermore, the plasma LPS level was significantly increased by 13.45% (*p* < 0.05) in the SVP group, and this increase was also significantly attenuated by *A. muciniphila* MucT.

### 3.4 Effect of sodium valproate on *Akkermansia muciniphila* level in vivo and in vitro

**FIGURE 4:**
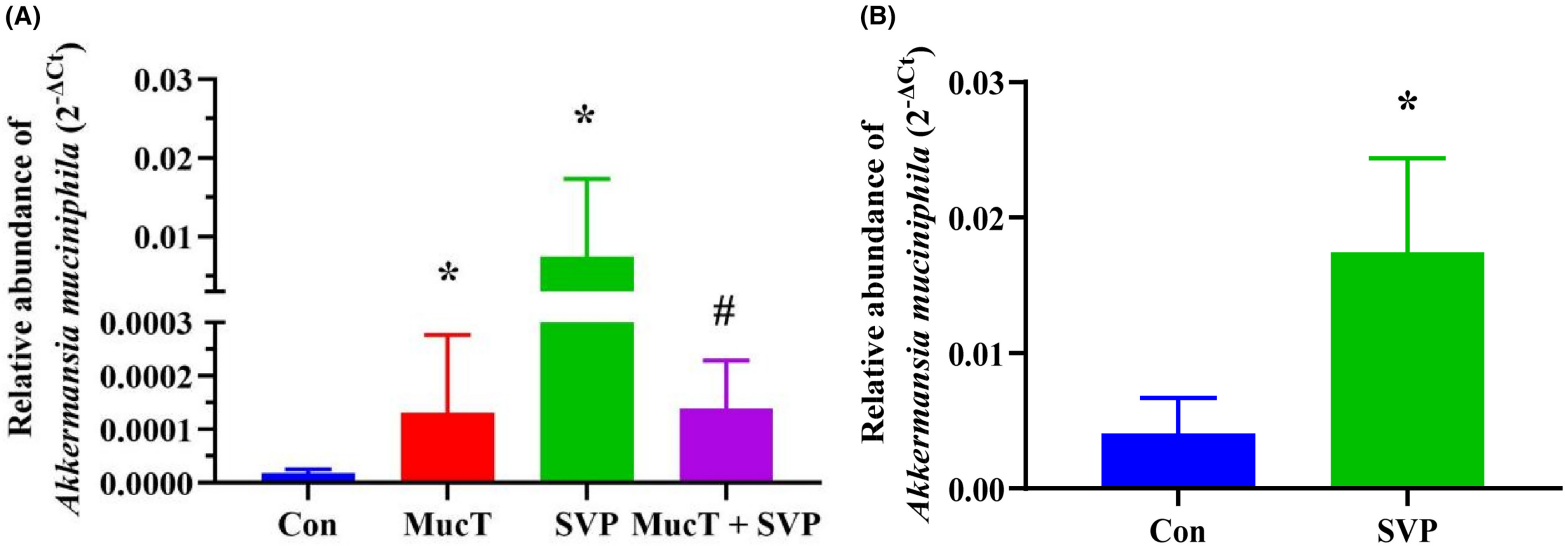
(A) Effect of sodium valproate alone and combined with *Akkermansia muciniphila* MucT on *Akkermansia muciniphila* level in vivo. Con: sterile anaerobic phosphate buffer solution; MucT: *A. muciniphila* MucT control group (10^8^ CFU in 1 mL sterile anaerobic phosphate buffer solution, once every other day, 9:00 AM.); SVP: 600 mg/kg SVP, once daily, 5:00 PM.; MucT + SVP: combined *A. muciniphila* MucT and SVP group. (B) Effect of sodium valproate on *Akkermansia muciniphila* level in vitro. Con: sterile anaerobic phosphate buffer solution; SVP: 10 mM SVP. 2^−ΔCt^, 2^−(Ct of *Akkermansia muciniphila* − Ct of total bacteria)^; Con, control; *A. muciniphila*, *Akkermansia muciniphila*; SVP, sodium valproate. *p < 0.05, compared with the control group; ^#^ *p* < 0.05, compared with the SVP group.

Compared with the control group, the administration of *A. muciniphila* MucT and SVP alone both led to a significant increase in the relative abundance of *A. muciniphila* in rats. Interestingly, the relative abundance of *A. muciniphila* in the combined *A. muciniphila* MucT and SVP group was significantly lower compared to the SVP group (Figure 4A).

**FIGURE 5:**
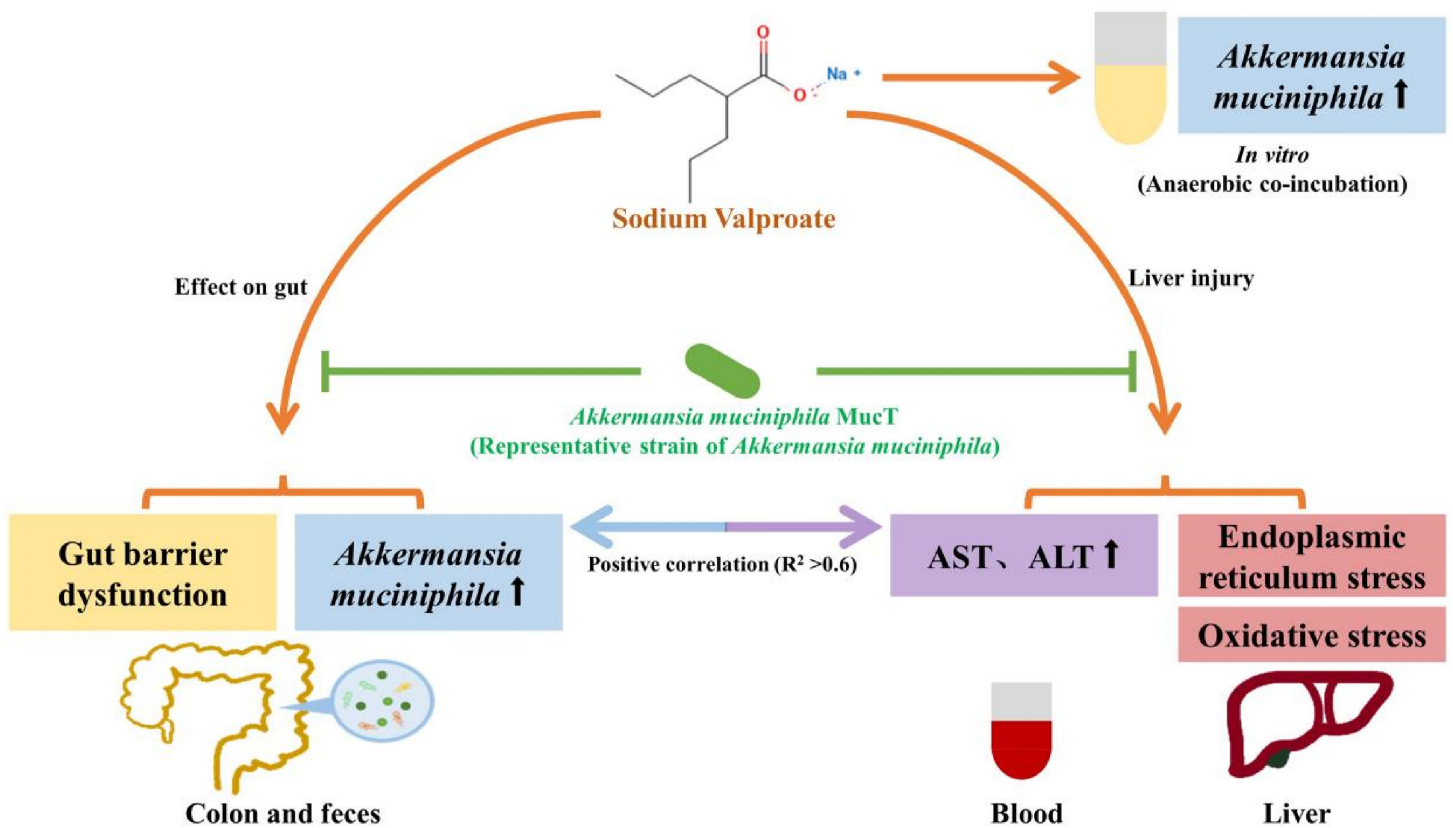
Schematic diagram of results in the study. Oral administration of *Akkermansia muciniphila* MucT attenuated sodium valproate‐induced hepatotoxicity and upregulation of *Akkermansia muciniphila* in rats. Sodium valproate treatment significantly increased the relative abundance of *Akkermansia muciniphila* in vivo and in vitro.

To further understand the role of *A. muciniphila* in SVP‐induced hepatotoxicity, we sought to investigate the effect of SVP on the relative faecal abundance of *A. muciniphila* in a host‐independent setting. Therefore, we carried out an experiment to anaerobically co‐incubate gut microbiota from faeces with SVP. The results demonstrated that SVP significantly up‐regulated the relative abundance of *A. muciniphila* by 4.30‐fold (*p* < 0.05), as demonstrated in Figure 4B.

## 4 DISCUSSION

The administration of SVP is associated with dose‐ and time‐dependent liver injury, even at clinically appropriate doses.^36^, ^37^ The exact molecular mechanism of SVP‐induced liver injury is not well understood. After decades of research, the hepatotoxicity of SVP has been widely speculated to be associated with toxic metabolites, oxidative stress, endoplasmic reticulum stress, mitochondrial damage, inhibition of fatty acid β‐oxidation and induction of fatty liver, etc.^38^ The gut microbiota is becoming a new direction for the exploration of the mechanism of drug‐induced liver injury. In our previous study, we observed that the relative faecal abundance of *A. muciniphila* increased after the oral administration of SVP, and there was a strong positive correlation between the relative abundance of *A. muciniphila* and liver function indicators ALT and AST activities (correlation coefficient >0.78).^15^ Because the opposite effects of *A. muciniphila* under different conditions have been reported, it is necessary to in‐depth investigate the effects of *A. muciniphila* in SVP‐induced hepatotoxicity. This study revealed that *A. muciniphila* MucT administration notably attenuated SVP‐induced hepatotoxicity. The favourable effect of *A. muciniphila* MucT administration on SVP‐induced hepatotoxicity was accompanied by the significant inhibition of oxidative stress and endoplasmic reticulum stress, and the recovery of gut barrier function. Moreover, the present study indicated that SVP treatment significantly increased the relative abundance of *A. muciniphila* in vivo and in vitro, and *A. muciniphila* MucT effectively reversed the increased relative abundance of *A. muciniphila* induced by SVP in vivo. Schematic diagram of results in the study is presented in Figure 5.

Oxidative stress is one of the common features of SVP‐induced liver injury,^38^ and anti‐oxidative stress is one of the important mechanisms by which *A. muciniphila* exerts hepatoprotective effect.^18^, ^20^ Oxidative stress results from an imbalance between oxidative and antioxidant effects, and is manifested by elevated levels of total oxidants and oxidation products, as well as reduced levels of endogenous antioxidants.^39^ Several studies have shown that SVP administration reduces enzymic and non‐enzymic antioxidants including SOD, CAT, GPx and GSH, and increases the indicators of peroxidation such as MDA (Indicator of membrane lipid peroxidation) and 8‐OHdG (Indicator of DNA peroxidation).^38^ The present results showed that SVP led to a significant decrease in SOD content and a significant increase in MDA and 8‐OHdG contents in rat liver. However, the endogenous antioxidant CAT, GPx and GSH levels in the liver of SVP‐administered rats showed only a tendency to decrease, which might be related to numerous factors such as sample size, dosage, etc. Nevertheless, these results still revealed that SVP induced oxidative stress. Compared with the SVP‐induced liver injury model group, the hepatic MDA content in the SVP co‐administrated with *A. muciniphila* MucT group was significantly reduced, and the SOD, CAT and GPx activities and GSH content also showed a tendency to recover. It was shown that *A. muciniphila* MucT attenuated SVP‐induced oxidative stress, especially lipid peroxidation, in rat liver tissues.

The endoplasmic reticulum is a determinant compartment for ensuring cell homeostasis through regulating the synthesis, processing and transport of most proteins. The redox imbalance can interfere with the endoplasmic reticulum function and trigger endoplasmic reticulum stress followed by an activation of apoptosis pathways.^40^ Endoplasmic reticulum stress is tightly linked to the liver injury of acetaminophen, isoniazid, SVP, etc.^41^ The Chop, Grp‐78 and caspase‐12 are important endoplasmic reticulum stress biomarkers.^42^ SVP administration significantly elevated the hepatic Chop, Grp‐78 and caspase‐12 protein levels, which was markedly inhibited by *A. muciniphila* MucT. These results indicated that anti‐endoplasmic reticulum stress is also an important mechanism for the protective effect of *A. muciniphila* MucT against SVP‐induced liver injury.

The gut barrier provides a first line of defence to protect our body from the damage of potentially toxicant, harmful bacteria and their antigens. The crypt‐villus architecture and mucus layer play a key role in the gut barrier function.^43^, ^44^ The colonic mucus layer is made up of the inner sublayer and outer mucus layer, and the former is renewed from the secretions of the goblet cells at the luminal border and expands distally as the latter.^45^ Therefore, the changes in colon mucosal thickness, crypt depth and goblet cell number were studied in the study through staining of tissue sections combined with image measurement. The colon mucosal thickness, crypt depth and goblet cell number were significantly reduced in rats after the SVP administration, and all these negative changes could be improved by *A. muciniphila* MucT. Bacteria‐derived LPS can act locally and cross the gut barrier followed by entering circulation, whose plasma concentration is a biomarker of gut barrier dysfunction.^46^ SVP administration resulted in a significant increase in plasma LPS level, and the plasma level of LPS in rats with SVP co‐administered with *A. muciniphila* MucT was close to that of normal control. These findings suggest that the maintenance of gut barrier function is one of the important mechanisms by which *A. muciniphila* MucT attenuates SVP‐induced liver injury.

The bidirectional communication between the gut, its microbiome and the liver known as the ‘Gut‐Liver axis’ has been the subject of extensive study. The liver is well‐known to exert a profound influence on immunity and metabolism in multiple organs, including the gut. Therefore, we could not rule out the possibility that the upregulation of faecal *A. muciniphila* level might be a result of SVP‐induced hepatotoxicity, not directly caused by SVP. In the present study, we conducted an in vitro study using anaerobic incubation of gut microbiota from faeces with SVP to investigate the effect of SVP on the relative abundance of *A. muciniphila* in the absence of host factors. The result of this experiment showed that the relative abundance of *A. muciniphila* increased following SVP treatment in vitro. In addition, *A. muciniphila* MucT administration significantly reversed the increased relative abundance of *A. muciniphila* induced by SVP in vivo. The above results suggested that this increase in the relative abundance of *A. muciniphila* could be directly induced by SVP. It is now widely accepted that the beneficial or harmful nature of a microbe is highly dependent on host, environment and microorganisms.^47^ Currently, there are 215 strains and 234 isolates of *A. muciniphila* retrievable from the NCBI database (https://www.ncbi.nlm.nih.gov/data‐hub/genome/?taxon=239935). It has been reported that *Akkermansia*‐affiliated 16S rRNA sequences derived from mammalian intestinal samples form five distinct clades.^48^ Different *A. muciniphila* genotypes exhibit different degrees and directions of change in relative abundance and exert diverse effects in the same disease.^49^ Therefore, we hypothesize that the increased *A. muciniphila* after the oral administration of SVP may differ from the representative strain of *A. muciniphila*, *A. muciniphila* MucT, used in this study. Further research is needed to verify this conjecture.

It is important to acknowledge some limitations of our study. Firstly, we have not isolated and identified the specific *A. muciniphila* strain that increased after SVP administration. This leaves unanswered questions about the potential impact of *A. muciniphila*, which was elevated after SVP administration, on the hepatotoxicity of SVP. Secondly, we could not investigate the effect of *A. muciniphila* on SVP‐induced hepatotoxicity using broad‐spectrum antibiotics to inhibit the upregulation of *A. muciniphila* as usually described in the literature.^27^ This was due to the administration of broad‐spectrum antibiotics (1 g/L ampicillin, 1 g/L neomycin, 1 g/L metronidazole, 1 g/L gentamicin and 0.5 g/L vancomycin) led to a significant increase in plasma LDH levels (Broad‐spectrum antibiotics vs control, 226.18 ± 64.21 U/L vs 123.26 ± 30.45 U/L, *p* = 0.0119) and an upward trend in plasma AST activities (Broad‐spectrum antibiotics vs control, 122.01 ± 28.26 U/L vs 94.22 ± 12.56 U/L, *p* = 0.0794). Finally, our study primarily aimed to elucidate the mechanisms underlying liver injury caused by SVP to promote safe drug use in clinical practice. Therefore, further investigation in a clinical setting is necessary to fully comprehend the role of *A. muciniphila* in this process.

## 5 CONCLUSION

Our study demonstrated that oral supplementation of *A. muciniphila* MucT effectively mitigated the hepatotoxicity induced by SVP in rats. Notably, the beneficial effects of *A. muciniphila* MucT were attributed, at least in part, to the reduction of oxidative stress and endoplasmic reticulum stress, and the maintenance of gut barrier function. These results suggest that oral supplementation of *A. muciniphila* MucT may be a potential therapeutic strategy for SVP‐induced hepatotoxicity, and further studies are warranted to investigate the potential clinical applications of these findings. Moreover, the present study indicated that SVP treatment significantly increased the relative abundance of *A. muciniphila* in vivo and in vitro, and *A. muciniphila* MucT effectively reversed the increased relative abundance of *A. muciniphila* induced by SVP in vivo. These findings provide new insights into the intricate interplay between *A. muciniphila* and SVP‐induced hepatotoxicity and suggest that different strains of *A. muciniphila* may confer distinct or even opposing effects on the development of SVP‐induced hepatotoxicity.

## AUTHOR CONTRIBUTIONS

**Zhi Cui:** Conceptualization (equal); data curation (equal); formal analysis (equal); investigation (equal); methodology (equal); writing – original draft (equal); writing – review and editing (equal). **Liang Xu:** Writing – review and editing (supporting). **Ming Zhao:** Conceptualization (equal); writing – review and editing (supporting). **Luping Zhou:** Conceptualization (equal); data curation (equal); formal analysis (equal); funding acquisition (equal); investigation (equal); methodology (equal); writing – original draft (equal); writing – review and editing (equal).

## CONFLICT OF INTEREST STATEMENT

The authors confirm that there are no conflicts of interest.

## Data Availability

Data will be made available on request.
